# The Influence of Teacher–Student Interaction on the Effects of Online Learning: Based on a Serial Mediating Model

**DOI:** 10.3389/fpsyg.2022.779217

**Published:** 2022-03-16

**Authors:** Hai-Long Sun, Ting Sun, Feng-Yi Sha, Xiao-Yu Gu, Xin-Ru Hou, Fei-Yan Zhu, Pei-Tao Fang

**Affiliations:** School of Business, Guangdong University of Foreign Studies, Guangzhou, China

**Keywords:** online education, teacher–student interaction, learning engagement, learning effect, chain-mediating effect

## Abstract

During the COVID-19 pandemic, online education has become an important approach to learning in the information era and an important research topic in the field of educational technology as well as that of education in general. Teacher–student interaction in online education is an important factor affecting students’ learning performance. This study employed a questionnaire survey to explore the influence of teacher–student interaction on learning effects in online education as well as the mediating role of psychological atmosphere and learning engagement. The study involved 398 college students studying at Chinese universities as the research object. Participants filled out a self-report questionnaire. The study found that (1) the level of teacher–student interaction positively affected students’ learning effects (*r* = 0.649, *p* < 0.01). (2) The psychological atmosphere mediated the positive effect of the level of teacher–student interaction on learning effects with mediating effect value of 0.1248. (3) Learning engagement mediated the positive effect of teacher–student interaction on learning effects with a mediating effect value of 0.1539. (4) The psychological atmosphere and learning engagement play a chain-mediating role in the mechanism of teacher–student interaction affecting students’ learning effects; that is, teacher–student interaction promotes students’ learning engagement by creating a good psychological atmosphere, which, in turn, influences learning effects. The mediating effect value was 0.0403. The results indicate that teacher–student interaction not only directly affects students’ learning effects but also influences students’ learning effects through the mediating effect of the psychological atmosphere and learning engagement.

## Introduction

The global spread of COVID-19 has resulted in the suspension of classes for more than 850 million students worldwide, disrupting schools’ original teaching plans in these countries and regions (Chen et al., 2020). Meanwhile, the update and development of network information technology has accelerated the digitalization process of traditional education, promoted the deep integration of subject courses and information technology, and promoted the practice and exploration of online education (Paudel, 2021). Many countries began offering online teaching to students *via* platforms, such as Zoom, Skype, and FaceTime. Today, online education has become a common form of learning that is affected by COVID-19. Based on the current situation of global epidemic prevention and control, online education is expected to be a long-standing teaching method (Moore et al., 2010; Chen et al., 2020).

In addition, past studies have primarily focused on traditional classroom contexts and merely extended the characteristics and regularity findings of traditional classrooms to online classroom studies. However, whether their findings can be applied to higher education in general or even higher education in online classrooms needs to be explored in depth. For instance, Carter and Rukholm (2008) speculated that, compared to traditional education, teacher–student interaction in online education is an important factor influencing students’ learning effects. How, then, do teachers and students interact effectively in online education in the era of COVID-19? How can learning effects be improved through teacher–student interaction? This is an important scientific and practical problem that must be solved urgently in online education. Based on this need, this study constructs a chain mediation model to explore the influence of teacher–student interaction on learning effects in online classrooms and determine what mediating factors of teacher–student interaction impact learning effects. Moreover, it provides a theoretical basis for relevant research and online teaching practice and has academic research and practical value.

## Theoretical Review and Research Hypothesis

In teaching, teacher–student interaction behaviors, which refer to the process of interaction between teachers and students during classroom teaching through a variety of situations, forms, and contents, are diverse, giving full play to the parties’ personal characteristics (Van de Pol et al., 2010). From the perspective of interaction theory, Zhou (2003) defined teacher–student interaction behaviors it as a multiform, multi-content, and multi-latitude interaction process between teachers and students in a common situation. Accordingly, the essence of teacher–student interaction is a system of interaction that is multiform and multi-content. Based on the concept of teacher–student interaction, we think that teacher–student interaction in online education refers to the process that contributes to teaching and learning in the context of online teaching, in which teachers and students play their roles and use Internet tools.

### The Level of Teacher–Student Interaction Affects Students’ Learning Effect

The level of teacher–student interaction improves students’ learning effects on two levels: interactive form and interactive content. In the form of teacher–student interaction, Moore (1989) proposed that online learning interaction includes three types of interactions: “learners and learning content,” “learners and teachers,” and “learners and learners.” On this basis, Li et al. (2020) further clarified that “Internet + teaching” is the “information interaction between teachers and students and teaching elements” in a specific environment, reflecting the change from one-way to multi-directional interaction. They also pointed out that the level of interaction is positive. This level is reflected in the quality of classroom questions. Studies have shown that the proportion of high-level questions that can bring better learning effects to classroom questions has increased significantly (Graesser and Olde, 2003).

At the level of teacher–student interactive content, multiple indicators, such as knowledge acquisition, ability training, emotional edification, and value establishment, constitute an interactive content system. Yang (2002) noted that effective learning activities are one of the basic conditions for learning to occur. Through the design and implementation of effective learning activities, an active learning process will occur, and better learning results will be achieved. Furthermore, some researchers have pointed out that effective teacher–student interaction is a necessary condition for deep learning in the context of online education (Mu and Wang, 2019); it is the strongest factor in the online learning experience (Jiang et al., 2019), and it is people who play a decisive role in the interaction between teachers and students. The effect of various interactive strategies in distance education is based on the joint efforts of teachers and students (Liu, 2006). As a result, this research proposes Hypothesis 1:

*H1: The level of teacher–student interaction is positively correlated with learning effects in online education.*

### The Mediating Role of Psychological Atmosphere

Social interaction theory refers to the process by which individuals take social actions toward others and each other and engage in reactive social actions; it emphasizes interactive behaviors that take place in specific contexts that have an impact on the psychology and behavior of both parties (Bandura, 1967). Furthermore, the influence of social interaction often needs to be realized through environmental changes (Seabi, 2012). Focusing on the teacher–student interaction perspective, we inferred that the degree of teacher–student interaction in online education *via* a good learning atmosphere improves the level of students’ participation in learning (i.e., the degree of learning investment), so as to promote learning effects. According to constructivism, the learner’s knowledge is obtained in a certain context with the help of others, using necessary information, as well as through the construction of meaning; the ideal psychological atmosphere should include context, collaboration, conversation, and meaning construction (He, 1997). Class atmosphere is a factor that affects individual achievement goals. The learning environment may focus on mastery, effort, or performance and ability, which affects the goal positioning of different individuals (Ames and Archer, 1988). Successful teaching is the result of the combined effect of variables, such as teachers, students, content, family, school, society, region, history, and culture (Pham et al., 2012). This research groups the selection of multiple variables into the “psychological atmosphere” as an important mediating variable in the influence mechanism of teacher–student interaction. Good two-way communication between teachers and students can shorten the psychological distance between the two as well as among students and encourage students to form a positive collective atmosphere (Tang and Zhong, 2013). Specific to live teaching courses, a teacher’s live streaming investment significantly affects the online psychological atmosphere (Yuan and Qi, 2020).

Furthermore, Zhang et al. (2020) found that a good classroom atmosphere is conducive to improving the teaching effect when studying classroom delivery. The classroom atmosphere affects students’ subjective environmental cognition, and students’ perception of the learning environment has an important impact on their academic performance (Yu et al., 2013). Combined with the findings of the above research, this research suggests that a good psychological atmosphere can enable students who are not directly supervised and are receiving online education to participate more actively in interaction with teachers and insert themselves into class learning, which helps students quickly enter a learning state in the classroom, and ultimately achieve a high-level learning effect. Therefore, Hypothesis 2 is further proposed:

*H2: The psychological atmosphere has a mediating effect between teacher–student interaction level and learning effects in online education.*

### The Mediating Role of Learning Engagement

Wilson (2006) summarized the conceptual model of three-dimensional learning engagement: behavior input, learning emotional input, and learning cognitive input. In addition, Shi (2010) suggested two important characteristics of students’ learning engagement: the effectiveness of the input and the student’s satisfaction with their learning status and school conditions. Effectiveness can be observed through GPA in the short term, and teacher–student interaction affects students’ satisfaction with online classrooms. Therefore, we believe that to achieve learning goals, teachers need to play a variety of roles in the classroom. The dialog between teachers and students, student feedback, and teacher evaluation are concrete manifestations of this process (Zhang, 2015). Thus, this study proposes Hypothesis 3:

*H3: The level of engagement has a mediating effect between teacher–student interaction level and learning effects in online education.*

Combining the previous assumptions, this study considered that the psychological atmosphere and learning engagement may have a chain-mediating effect between teacher–student interaction and learning effect. In other words, teacher–student interaction → psychological atmosphere → learning engagement → learning effect. Research of Allen and Griffeth (2001) shows that multiple mediators exhibit sequential effects that form a chain of mediators, which is referred to as the chain-mediating model. Therefore, Hypothesis 4 is proposed as follows:

*H4: Psychological atmosphere and learning engagement have a chain-mediating effect between teacher–student interaction and learning effects in online education.*

**Figure 1:**
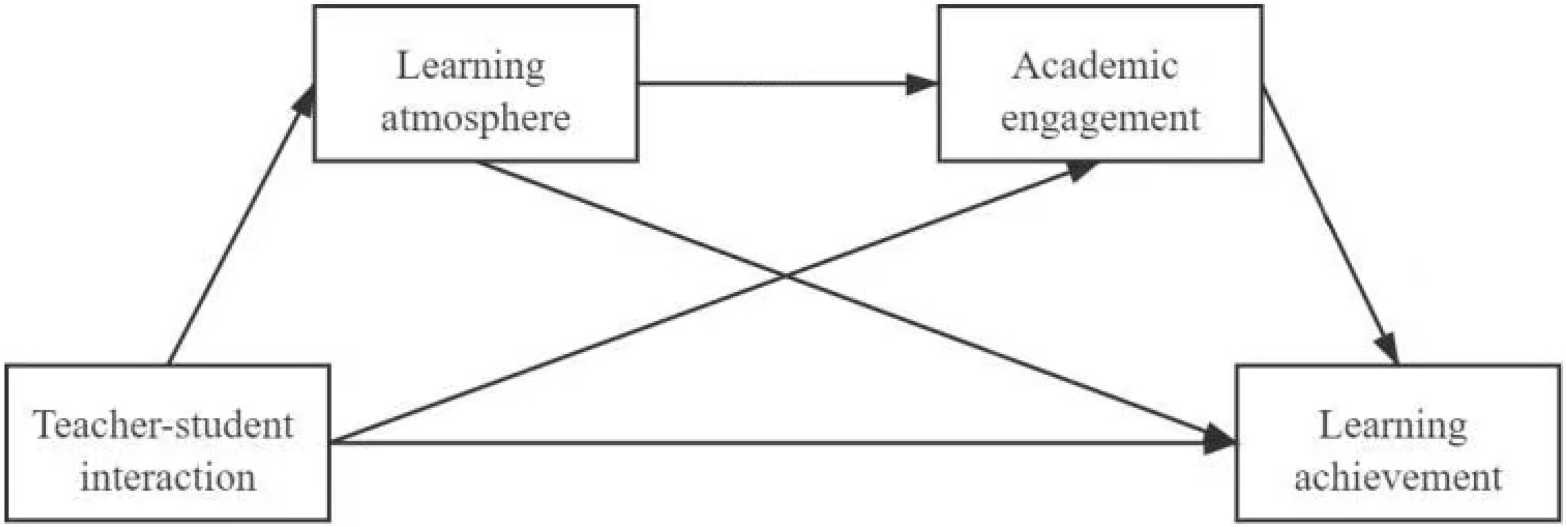
Hypothetical model of the effect of teacher–student interaction on learning effect in online education.

Based on the above analysis, a hypothetical model is proposed (see Figure 1).

## Materials and Methods

### Participants

In this study, a random sample of students of different grades, majors, places of origin, and types of schools who had participated in online education at the undergraduate level or above was included as participants. The investigated sample was recruited online *via* Wenjuanxing,^1^ an online platform similar to Mechanical Turk or Qualtrics, which is used to launch nationwide e-surveys in China and is widely employed in behavioral and psychological studies. Participants gave their informed consent after being provided with information explicitly stating the research purpose as well as the nature and procedure of the study. A total of 508 questionnaires were returned. As the quality of online questionnaires is difficult to guarantee, some cases chose the same response for the entire questionnaire. Questionnaires with more than three standard deviations were excluded. An effective total of 398 participants was obtained (180 males, 218 females; Table 1). When the target population increases, researchers must gradually increase the sample size. Existing studies suggest that when the target population reaches 5,000 or above, the sample size can be increased to approximately 350–500, which indicates that our sample size of 398 is sufficient.

**Table 1:** Demographic characteristics of the sample.

Variable	Category	Frequency (n)	Percentage
Gender	Male	180	45.2
	Female	218	54.8
Course type	Skill	198	49.7
	Theory	200	50.3
Grade	Freshman	29	7.3
	Sophomore	156	39.2
	Junior	205	51.5
	Senior	8	2.0
University type	“985” university	33	8.3
	“211” university	27	6.8
	Ordinary university	338	84.9

985 universities are universities at the first level on the Chinese mainland. 211 universities refer to better universities in China (100 key universities in the 21st century: 211).

### Measurement

#### Teacher–Student Interaction Scale

The Teacher–Student Interaction Scale was revised according to Xu (2016) and used to measure the level of teacher–student interaction using a 5-point Likert scale. Each item was rated from 1 (strongly disagree) to 5 (strongly agree), with higher scores indicating higher teacher–student interaction. The scale includes six dimensions—interaction quantity, interaction form, interaction distance, interaction content, interaction time, and interaction motivation—with a total of six items, such as “In online education, I can speak freely in class.” *Cronbach’s alpha* was 0.830.

#### Learning Engagement Scale

The Learning Engagement Scale was revised from the classroom engagement scale developed by Wang et al. (2014) to measure students’ engagement in the classroom using a five-point Likert scale ranging from 1 (strongly disagree) to 5 (strongly agree). This scale has three items, including “I can solve problems using multiple solutions in online education.” Cronbach’s alpha coefficient was 0.785 in this study.

#### College Student Classroom Psychological Atmosphere Scale

Learning atmosphere was measured using the Psychological Atmosphere Scale developed by Li (2006, unplubished) to measure the atmosphere of college classes. It uses a 5-point Likert scale and consists of five items, each rated from 1 to 5 (1 = strongly disagree, 5 = strongly agree). Higher scores indicate a higher-quality psychological atmosphere. In this study, we used the learning and collaboration dimensions of scale of Li and additionally developed a psychological atmosphere subscale with five questions, taking into account the characteristics of online education, for example, “the teacher is highly concerned with classmates.” In this study, Cronbach’s alpha coefficient for the Psychological Atmosphere Scale was 0.795.

#### Learning Effect Scale

The learning evaluation system under the mixed education model was developed by Zhou (2018) for multidimensional dynamic learning evaluation using a five-point Likert scale. This study’s learning effect scale adopted the three dimensions of online independent learning, offline collaborative learning, and classroom interactive learning in the “learning evaluation system under mixed education model” and additionally developed a subscale that combined the actual evaluation criteria of university courses, which contains seven keywords: concentration, duration, initiative, cooperation, satisfaction, communication, and application. For example, “I discovered that my interest in learning has improved significantly.” Cronbach’s *α* coefficient for the Learning Engagement Scale was 0.910 in this study.

#### Control Variables

To control for the influence of other factors, we also measured the participants’ gender and grade and the types of courses as control variables.

### Study Procedure

In this study, data were collected using a time-lag design to avoid common method bias. Specifically, the data collection in this study was divided into three time points with a 1-week interval. Data were collected *via* participants’ self-reporting. Materials were prepared in Chinese and presented in a questionnaire form. At the first time point, we collected independent variables (degree of interaction) and control variables; at the second time point, we collected intermediary variables (psychological atmosphere, learning engagement); finally, at the third time point, we collected dependent variables (learning effects in online education).

## Results

Compared to other statistical methods (e.g., regression analysis or structural equation modeling), the bootstrap method is suitable for small samples and does not assume a data distribution morphology. Therefore, for the purpose of this study, the bootstrap method proposed by Preacher and Hayes (2004, 2008) was used to examine mediation effects.

In this study, SPSS24.0 and AMOS software were employed to test common method bias as well as the reliability of the analysis results. Additionally, SPSS24.0 was used to perform a descriptive statistical analysis of the variables, including calculating their mean, the standard deviation, the measured reliability coefficient, and the correlation coefficients between the variables. Then, for the unstandardized scale means, we performed the chain mediation effect test using the PROCESS macro test in SPSS 24.0.

### Common Method Biases

Based on the completion of the exploratory factor analysis, this study continued the validation factor analysis using the common method bias analysis method: all items of the four variables of psychological atmosphere, interaction level, engagement level, and learning effect were evaluated using exploratory factor analysis. A common method factor was then added, and a one-way validating factor analysis was performed with all the scale items as those involved in the hypothesis testing. The results showed that *ΔRMSEA* = 0.011, *ΔSRMR* = 0.0147 < 0.05, *ΔCFI* = 0.035, and *ΔTLI* = 0.032 < 0.1. This shows that after the common method factor is added, there is no significant common method deviation, and the model has good discriminative validity.

### Correlation Analysis

The descriptive statistics and correlation matrix for each variable are listed in Table 2. The results showed that psychological atmosphere, degree of interaction, learning engagement, and learning effect are significantly positively correlated at the 1% level, indicating that further mediation effects can be tested. There was a positive correlation between psychological atmosphere and degree of interaction (*r* = 0.606, *p* < 0.01), learning engagement and learning effects (*r* = 0.640, *p* < 0.01), and degree of interaction and learning effects (*r* = 0.649, *p* < 0.01). Psychological atmosphere and learning engagement (*r* = 0.406, *p* < 0.01), psychological atmosphere and learning effects (*r* = 0.566, *p* < 0.01), and degree of interaction and learning engagement were positively correlated (*r* = 0.493, *p* < 0.01).

**Table 2:** Correlation coefficient table of research variables.

Variables	*M*	*SD*	1	2	3
1. Psychological atmosphere	3.366	0.658	1		
2. Degree of interaction	3.349	0.641	0.606^**^	1	
3. Learning engagement	3.456	0.654	0.406^**^	0.493^**^	1
4. Learning effects	3.526	0.641	0.566^**^	0.649^**^	0.640^**^

*M* (Mean), Arithmetic mean; SD, standard error of the mean.

** *p* < 0.01.

### Analysis of Control Variables

First, we discuss the factors that may influence the findings (grade and course type). Due to the large difference in numbers, we combined the freshman and sophomore numbers as the lower-grade group and the junior and senior grades as the senior group. Independent sample *t*-test results showed that compared to the higher grades (3.38 ± 0.65), the degree of learning engagement in the lower grades (3.51 ± 0.65) was significantly higher [*t*(396) = 1.95, *p* = 0.05]. However, there was no significant difference in the psychological atmosphere, degree of interaction, or learning effects between the higher and lower grades. Similarly, we analyzed the differences between the different types of courses. The results showed that there was no significant difference in the psychological atmosphere, degree of interaction, learning effects, or learning engagement between skill courses and theory courses. In addition, there was no significant correlation between the type of course and the variables (psychological atmosphere, degree of interaction, learning engagement, and learning effects).

### Analysis of Chain Mediating Effect

Mediation analyses were performed using the bootstrapping method with bias-corrected confidence estimates for the mediating effect of teacher–student interaction and student learning effects after controlling for gender, grade, and major as covariate variables (Preacher and Hayes, 2004).

First, based on the results in Table 3, teacher–student interaction has a significant impact on the learning effect (*β* = 0.331, *t* = 7.53, *p* < 0.001). After the mediating variables are included, learning engagement has a significant positive impact on the learning effect (*β* = 0.390, *t* = 10.47, *p* < 0.01), and psychological atmosphere not only has a significant positive impact on the learning effect of students (*β* = 0.201, *t* = 4.97, *p* < 0.001) but also has a moderately significant impact on learning engagement (*β* = 0.168, *t* = 3.10, *p* < 0.005). The level of teacher–student interaction not only positively affects the psychological atmosphere of students (*β* = 0.623, *t* = 15.01, *p* < 0.001) and learning engagement (*β* = 0.402, *t* = 7.18, *p* < 0.001) but also has a positive effect on learning effects. Significant positive effects were observed (*β* = 0.331, *t* = 7.53, *p* < 0.001). Gender, grade, and major as controlled variables all had *p*-values greater than 0.1 (specified ^*^p < 0.05, ^**^p < 0.01), indicating that all three had a small effect on all four dimensions, with negligible effects in terms of the chain-mediating effect.

**Table 3:** Regression analysis of the mediation model.

Variable	Model 1: Learning effect		Model 2: Psychological atmosphere		Model 3: Learning engagement		Model 4: Learning effect	
	*β*	*t*	*β*	*t*	*β*	*t*	*β*	*t*
Gender	0.001	0.01	−0.076	−1.11	−0.065	−0.88	0.046	0.85
Grade	0.033	0.96	−0.003	−0.09	−0.014	−0.35	0.040	1.34
Course	−0.003	−0.19	−0.021	−1.20	0.017	0.91	−0.004	−0.30
Interactive	0.654	16.87	0.623	15.01	0.402	7.18	0.331	7.53
Atmosphere					0.168	3.10	0.201	4.97
Engagement							0.390	10.47
*R^2^*	0.423		0.371		0.266		0.59	
*F*	72.07		57.85		28.36		92.33	

*β: Regression coefficient; T: The result of the t-test on the regression coefficient. The mediating effect analysis (see* Table 4*) shows that the Bootstrap 95% confidence interval of the mediating effect of psychological atmosphere and learning engagement does not contain 0, indicating that psychological atmosphere and learning engagement are due to teacher–student interaction affecting student learning. Regarding the mediating variable of effect, the total mediating effect value was 0.319. Specifically, the mediating effect of teacher–student interaction on student learning is primarily achieved through the following three paths: (1) indirect effect 1 (0.1248): teacher–student interaction level → psychological atmosphere → student learning effect; (2) indirect effect 2 (0.1539): teacher–student interaction level → learning engagement level → learning effect; and (3) indirect effect 3 (0.0403): teacher–student interaction → psychological atmosphere → learning engagement level → learning effect. Indirect effect 1, indirect effect 2, and indirect effect 3 accounted for 19.20, 23.68, and 6.20% of the total effect, respectively. Indirect effect 2 was more significant than indirect effect 1, and indirect effect 1 was more significant than indirect effect 3, while other differences did not reach a significant level.*

**Table 4:** Bootstrap results for the mediation effect.

Mediating path	Indirect effect	Boot standard error	95% confidence interval		Relative mediation effect	Total mediation effect
			Lower limit	Upper limit		
Total indirect effect	0.319	0.0464	0.2311	0.4134	100.00%	49.08%
Indirect effect 1	0.1248	0.0343	0.0622	0.1949	39.12%	19.20%
Indirect effect 2	0.1539	0.0382	0.083	0.2347	48.24%	23.68%
Indirect effect 3	0.0403	0.0186	0.0064	0.0795	12.63%	6.20%

**Figure 2:**
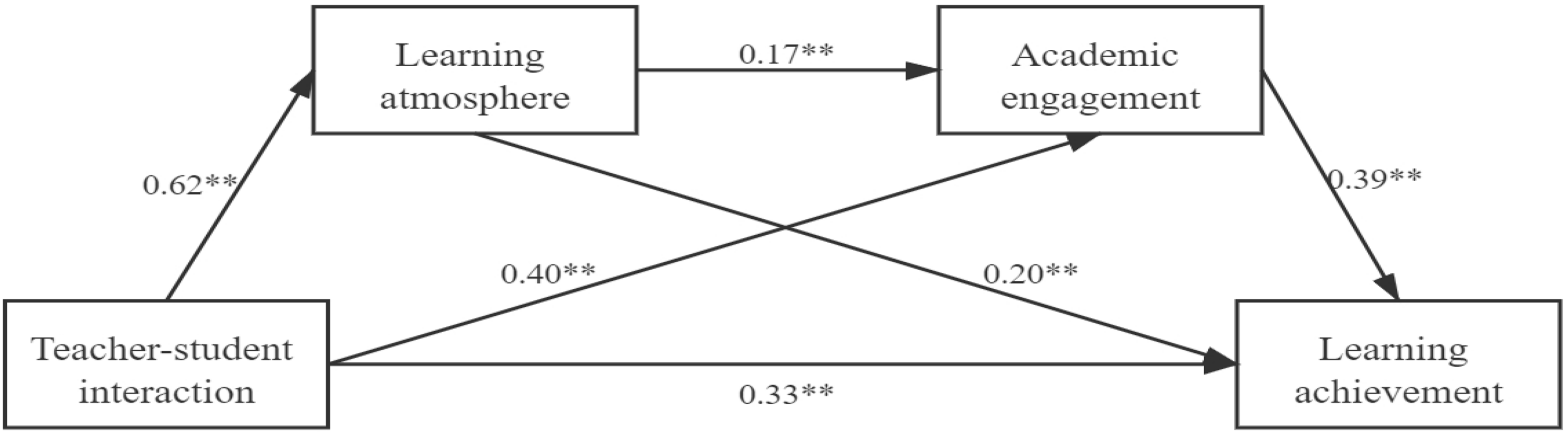
The chain mediation model of the influence of teacher–student interaction level on student learning effects in online education **p < 0.01.

The above results further support that the psychological atmosphere plays a mediating role between teacher–student interaction level and the effects of online learning, and learning engagement plays a mediating role between teacher–student interaction level and the effects of online learning for students. The level of teacher–student interaction and the effect of students’ online learning play a chain-mediating role (see Figure 2).

## Discussion

This research explores the impact of teacher–student interaction on learning effects in online education as well as the mediating effect of psychological atmosphere and learning engagement. The results show that teacher–student interaction not only positively influences learning effects but also has a positive impact on learning effects through the mediating effect of psychological atmosphere and learning engagement. In addition, psychological atmosphere and learning engagement have a chain-mediating effect on the influence mechanism of teacher–student interaction that affects learning effects for students. That is, teacher–student interaction promotes students’ learning engagement by creating a positive psychological atmosphere, which, in turn, affects the learning effects experienced by students. Therefore, this study concludes that the level of teacher–student interaction not only directly affects learning effects but also influences them through the mediating effect of psychological atmosphere and learning engagement and the chain-mediating effect of psychological atmosphere–learning engagement. Three research implications are drawn as follows: enhancing the level of teacher–student interaction can improve learning effects, enhancing online classroom atmosphere and learning engagement can strengthen student learning effects, and building a new type of teacher–student relationship can better promote student learning effects.

### The Level of Teacher–Student Interaction Affects the Learning Effects of Students Engaged in Online Education

Debourgh (2003) believed that teacher–student interaction is an important factor affecting the learning effects of students in online education. Lin et al. (2017) found that the interaction between learners and teachers has a significant positive impact on online learners’ learning satisfaction as well as on learning effects. The analysis in this research shows that the level of teacher–student interaction has a positive impact on learning engagement and psychological atmosphere. Therefore, this further demonstrates that the level of teacher–student interaction will affect the learning effects of students in online education from another perspective, which is consistent with existing research. However, the previous analysis shows that the level of teacher–student interaction has different degrees of influence on different mediators, in which teacher–student interaction has a greater impact on the academic atmosphere and students’ learning engagement as a whole, followed by learning effects. Teacher–student interaction has a significant positive effect on the above three mediators. Existing research shows that interaction in online learning is closely related to learners’ learning experience, learning engagement, learning satisfaction, and learning effects. For example, Zhang et al. (2017) found that in online learning, the multi-level interaction between students, teachers, and among students is beneficial for improving students’ learning effects. Therefore, how to improve the interaction level between teachers and students in online education to facilitate its impact on students’ learning effects should receive attention in the field of online education.

### The Chain Mediating Effect of Learning Engagement and Psychological Atmosphere

This research further reveals how teacher–student interaction level affects the learning effects of students through the mediators by dividing different dimensions of the mediators of the teacher–student interaction influence mechanism. Although they all have an impact, different mediators have different degrees of influence on students’ learning effects. Among them, the level of learning engagement as a mediator has the greatest impact on the learning effects of students, followed by psychological atmosphere. Tang (2018) also began by building teacher–student relationships to enhance students’ perceptions of good teacher–student relationships, improve the perceived school atmosphere, and promote students’ learning engagement. Our results of study not only expand the previous theoretical research of the teacher–student interaction level–student learning effect and demonstrate the positive influence of the mediators of students’ psychological atmosphere and learning engagement level but also supplement and perfect the existing research on the influence mechanism of the existing teacher–student interaction level on the students’ learning effects. This is of great significance for improving students’ learning performance in online education by designing teacher–student interaction in the future.

### Practical Implications

Enhancing teacher–student interaction level can improve students’ learning effects. Teacher–student interaction plays a positive role in mobilizing the classroom atmosphere, guiding students to form correct learning attitudes, and improving learning effects (Van de Pol et al., 2010). In higher education, teacher–student interaction enhanced. For example, teachers should add more interactive sessions and release classroom learning evaluation results in a timely manner to improve the synchronous interaction between teachers and students, which can help students reflect on their performance in class discussion, improve their learning attitude and methods, and enhance their learning performance.

Research has found that improving the psychological atmosphere and increasing the level of student engagement in online education can help enhance the learning effects of online education. Based on this mechanism, in the online education learning process, the design of teacher–student interaction achieves the purpose of improving students’ learning performance and maximizing students’ learning effects by improving the psychological atmosphere, thereby increasing students’ learning enthusiasm and learning engagement.

In addition, it has been found that spiritual communication and the exchange of ideas between teachers and students are needed to foster harmonious development for both parties so as to achieve better teaching results (Pennings et al., 2018). Teacher–student interaction is also a reflection of the relationship between teachers and students. Teachers and students must communicate emotionally to form spiritual interactions and build a new type of interactive teacher–student relationship. By adopting cooperative teaching, teachers and students can establish a sharing mechanism to better promote the improvement of students’ learning effects.

### Research Limitations and Prospects

This research provides a theoretical contribution and practical value in regard to research on the influence mechanism of teacher–student interaction level on students’ online learning effect. However, has several limitations.

First, in terms of questionnaire design, due to the lack of control questions in the questionnaire, there is no question specifically used to identify whether the respondent answered the questionnaire seriously and truthfully. Therefore, there may be invalid questionnaires and data deviations. In the analysis portion of the questionnaire, there may be objective factors that have not been accounted for, which must be addressed in the future. In terms of research methods, the questionnaire survey method is a sample survey; thus, there may be individual differences in reality.

Second, although this study examines the effects of two types of intermediary variables, learning atmosphere and learning engagement, on the level of teacher–student interaction and student learning effects, the relationship between the two may also be affected by other factors. Therefore, future research should explore the boundary conditions that impact the effect of teacher–student interaction on students’ learning effects.

Third, there may be other factors that influence the results. Although factors, such as gender, grade, and type of course, were measured and treated as control variables in our study, the potential influences of other factors, such as the learner’s initial level of competence in the subject studied, cannot be ruled out. Future research should conduct separate analyses for different disciplines.

Fourth, the variety of university courses is rich, and course teaching is often not cohesive. For example, the students involved in this research were taking online education courses, and they will not be studied again before or after the semester of the study period. Therefore, considering the number of research samples and the rich variety of courses, we chose individual self-evaluations to measure learning effects. We believe that third-party evaluation is also a useful indicator for providing more objective data. Therefore, the matching samples can be adopted in future studies, in which teachers can evaluate learning effects or the interaction degree of their students to increase the objectivity and accuracy of the results.

Regarding future research, as people have continually paid attention to interaction, and interactive media with strong interaction capabilities prompts people to seek solutions to problems from a technical level, deep and effective interaction cannot be guaranteed.

With the continuous development of online education, teaching, and learning conditions will continue to change. Therefore, we should identify and pay close attention to these changing conditions over time and further restrict the research scope based on the new conditions. Moreover, future research should divide teacher–student interaction into different dimensions, focus more on the construction of teachers’ dialog ability in online education, avoid technicism, focus on inducing deeper learning for higher-level progress, and deeply explore the level of teacher–student interaction in a universal interaction framework. Finally, the specific influencing factors of students’ online learning effects should be examined, ineffective or inefficient teacher–student interactions should be explored from multiple perspectives, and feasible countermeasures and suggestions should be identified to improve the quality of teacher–student interaction and the effects of students’ online learning to further promote “teaching and learning” and fundamentally improve the quality of online education.

## Data Availability Statement

The original contributions presented in the study are included in the article/supplementary material, and further inquiries can be directed to the corresponding author.

## Ethics Statement

The studies involving human participants were reviewed and approved by the ethical standards of institutional review board at Guangdong University of Foreign Studies. The patients/participants provided their written informed consent to participate in this study.

## Author Contributions

H-LS and TS: conceptualization, writing— review, and editing. H-LS, TS, and F-YS: methodology. X-RH, F-YZ, and X-YG: formal analysis. H-LS, F-YZ, F-YS, and P-TF: writing—original draft preparation. H-LS: supervision. All authors contributed to the article and approved the submitted version.

## Funding

This research was partially supported by the National Natural Science of China (No.72101062), Guangdong Basic and Applied Basic Research Foundation (No. 2020A1515110429), Youth Foundation of Social Science and Humanity, China, Ministry of Education (No. 20YJCZH135), Youth Project of Social Science Foundation of Guangdong Province (No. GD19YGL07), and Education reform project of Guangdong University of Foreign Studies (GUDFS (2019), No. 55).

## Conflict of Interest

The authors declare that the research was conducted in the absence of any commercial or financial relationships that could be construed as a potential conflict of interest.

## Publisher’s Note

All claims expressed in this article are solely those of the authors and do not necessarily represent those of their affiliated organizations, or those of the publisher, the editors and the reviewers. Any product that may be evaluated in this article, or claim that may be made by its manufacturer, is not guaranteed or endorsed by the publisher.

## Footnotes

^1^ http://www.wjx.com

## Appendix

1. Teacher–student Interaction Scale

1. I can speak freely in class in online education.
2. Teacher–student interaction is getting longer in online education.
3. In online education, I prefer to interact with the teacher synchronously.
4. In online education, I will take the initiative to raise my hand to answer when I have an idea.
5. My classmate has become more interested in online education.
6. In online education, students in our class actively participate in classroom interaction.

2. Learning Engagement Scale

1. In online education, I can complete basic exercises and do extended exercises.
2. In online education, I can solve many problems with divergent and comprehensive thinking abilities.
3. In online education, I can solve problems through multiple solutions.

3. College Student Classroom Psychological Atmosphere Scale

1. In online education, many students are participating in the interaction in class.
2. I have participated in many teacher–student interaction courses in online education.
3. Teachers in online education provide many opportunities for interaction.
4. The forms of teacher–student interaction in online education have become diverse.
5. The timeliness of teacher–student interaction in online education has been improved.

4. Learning Effect Scale

1. Good teacher–student interaction in online education can improve my interest in learning.
2. Good teacher–student interaction in online education keeps me from getting distracted in class.
3. Good teacher–student interaction in online education can improve my learning hours after class.
4. In online education, I can listen carefully in class, do homework seriously, and actively participate in discussions.
5. I am good at cooperating with others in online education, not only having an opinion, but also listening to others’ opinions with an open mind.
6. Good teacher–student interaction in online education can improve my satisfaction with the course.
7. Good teacher–student interaction in online education can improve my professional communication skills.
8. Good teacher–student interaction in online education enables me to better use the knowledge of the major.

## References

[ref1] Allen D. G. Griffeth R. W. (2001). Test of a mediated performance-turnover relationship highlighting the moderating roles of visibility and reward contingency. J. Appl. Psychol. 86, 1014–1021. doi: 10.1037/0021-9010.86.5.1014, PMID: 11596795

[ref2] Ames C. Archer J. (1988). Achievement goals in the classroom: students’ learning strategies and motivation processes. J. Educ. Psychol. 80, 260–267. doi: 10.1037/0022-0663.80.3.260

[ref3] Bandura A. (1967). Social Learning Theory. Englewood Cliffs, NJ: Prentice Hall.

[ref4] Carter L. M. Rukholm E. (2008). A study of critical thinking, teacher-student interaction, and discipline-specific writing in an online educational setting for registered nurses. J. Contin. Educ. Nurs. 39, 133–138. doi: 10.3928/00220124-20080301-03, PMID: 18386701

[ref5] Chen T. Peng L. Yin X. Rong J. Yang J. Cong G. (2020). Analysis of user satisfaction with online education platforms in China during the COVID-19 pandemic. Healthcare 8:200. doi: 10.3390/healthcare8030200, PMID: 32645911 PMC7551570

[ref6] Debourgh G. A. (2003). Predictors of student satisfaction in distance-delivered graduate nursing courses: what matters most? J. Prof. Nurs. 19, 149–163. doi: 10.1016/S8755-7223(03)00072-3, PMID: 12836145

[ref8] Graesser A. C. Olde B. A. (2003). How does one know whether a person understands a device? The quality of the questions the person asks when the device breaks down. J. Educ. Psychol. 95, 524–536. doi: 10.1037/0022-0663.95.3.524

[ref9] He K. K. (1997). The teaching mode, teaching method, and teaching design of constructivism. J. Beijing Nor. Uni. 5, 74–81.

[ref10] Jiang Y. J. Bai X. M. Wu W. C. Luo X. J. (2019). Analysis of the structural relationship of influencing factors of the online learning experience. Mod. Distance Educ. 1, 27–36. doi: 10.13927/j.cnki.yuan.2019.0004

[ref12] Li X. W. Ye W. J. Zhang Q. H. (2020). Research on the index model of teacher-student interaction behavior in the environment of “internet+teaching”. Res. High. Educ. Eng. 3, 157–162.

[ref13] Lin C. H. Zheng B. Zhang Y. (2017). Interactions and learning outcomes in online language courses. Br. J. Educ. Technol. 48, 730–748. doi: 10.1111/bjet.12457

[ref14] Liu X. (2006). On the principles of teacher-student interaction in distance education from the perspective of dialogue theory. Distance Educ. China 3, 37–39.

[ref15] Moore M. G. (1989). Three types of interaction. Am. J. Dist. Educ. 2, 1–6. doi: 10.1080/08923648909526659

[ref16] Moore J. L. Dickson-Deane C. Galyen K. (2010). Designing for E-learning, online learning, and distance learning environments: are they the same? Internet High. Educ. 2, 129–135.

[ref17] Mu S. Wang X. J. (2019). Research on deep learning strategies in online learning. Distance Educ. China 10, 29–39+93.

[ref18] Paudel P. (2021). Online education: benefits, challenges, and strategies during and after COVID-19 in higher education. Int. J. Stud. Educ. 3, 70–85. doi: 10.46328/ijonse.32

[ref19] Pennings H. J. Brekelmans M. Sadler P. Claessens L. C. van der Want A. C. van Tartwijk J. (2018). Interpersonal adaptation in teacher-student interaction. Learn. Instr. 55, 41–57. doi: 10.1016/j.learninstruc.2017.09.005

[ref20] Pham G. Koch T. Helmke A. Schrader F. Helmke T. Eid M. (2012). Do teachers know how their teaching is perceived by their pupils? Procedia Soc. Behav. Sci. 46, 3368–3374. doi: 10.1016/j.sbspro.2012.06.068, PMID: 32712931

[ref21] Preacher K. J. Hayes A. F. (2004). SPSS and SAS procedures for estimating indirect effects in simple mediation model. Behav. Res. Methods Instrum. Comput. 36, 717–731. doi: 10.3758/BF03206553, PMID: 15641418

[ref22] Preacher K. J. Hayes A. F. (2008). Asymptotic and resampling strategies for assessing and comparing indirect effects in multiple mediator models. Behav. Res. Methods 40, 879–891. doi: 10.3758/BRM.40.3.879, PMID: 18697684

[ref23] Seabi J. (2012). Feuerstein’s mediated learning experience as a vehicle for enhancing cognitive functioning of remedial school learners. Aust. J. Educ. Dev. Psychol. 12, 35–45.

[ref24] Shi F. H. (2010). A new perspective on quality evaluation reform of undergraduate education: learning engagement. Mod. Educ. Manag. 5, 51–54. [in Chinese]. doi: 10.3969/j.issn.1674-5485.2010.05.015

[ref25] Tang C. L. (2018). The relationship between perceived school climate and learning engagement in junior middle school students and intervention study. Doctoral dissertation. Central China Normal University. [in Chinese].

[ref26] Tang S. G. Zhong W. L. (2013). Research on the construction of interactive teaching model. Theory Pract. Educ. 18, 42–43.

[ref27] Van de Pol J. Volman M. Beishuizen J. (2010). Scaffolding in teacher-student interaction: a decade of research. Educ. Psychol. Rev. 22, 271–296. doi: 10.1007/s10648-010-9127-6

[ref29] Wang Z. Bergin C. Bergin D. A. (2014). Measuring engagement in fourth to twelfth grade classrooms: the classroom engagement inventory. Sch. Psychol. Q. 29, 517–535. doi: 10.1037/spq0000050, PMID: 24708283

[ref30] Wilson T. D. (2006). On user studies and information needs. J. Doc. 62, 658–670. doi: 10.1108/00220410610714895

[ref001] Xu E. Q. (2016). An empirical study on the impact of teacher-students interaction on online learning performance. Res. Audio-Vis. Educ. 37, 61–68. doi: 10.13811/j.cnki.eer.2016.09.010

[ref31] Yang K. C. (2002). Several issues concerning the development of instructional design theory in the internet age. Mod. Educ. Technol. 1, 20–23+76.

[ref32] Yu H. Q. Li C. Shi M. M. (2013). The influence of learning environment on college students’ learning style and academic achievement: an empirical study based on the cultivation of undergraduate top innovative talents. J. High. Educ. 8, 62–70.

[ref33] Yuan Y. R. Qi S. J. (2020). A study on the influencing factors of college students’ learning ability in online teaching environment. Educ. Rev. 8, 61–64.

[ref34] Zhang Z. P. (2015). Research on teacher-student interaction in foreign classrooms: hot issues and future trends. Pri. Seco. Sch. Abr. 4, 42–48+41.

[ref002] Zhang W. P. Chen M. T. Zhao X. N. Bai X. (2020). Influence of teacher-student interaction on classroom learning in special delivery classroom: a case of art delivery class in chongyang primary school. Res. Audio-Vis. Educ. 41, 90–96. doi: 10.13811/j.cnki.eer.2020.08.012

[ref35] Zhang X. L. Huang Z. Z. Li M. L. (2017). An empirical study of online learners’ “interactive learning” experience and its impact on learning outcomes. Tsinghua J. Educ. 2, 117–124.

[ref36] Zhou Q. F. (2003). Constructing a language classroom of “teacher-student interaction”. Doctoral dissertation. East China Normal University.

[ref37] Zhou X. C. (2018). Exploration of assessment system and rating scales based on the blended learning mode. J. Chengdu Nor. Uni. 37, 163–167.

